# Privacy Implications of Contacting the At-Risk Relatives of Patients with Medically Actionable Genetic Predisposition, with Patient Consent: A Hypothetical Australian Case Study

**DOI:** 10.3390/biotech12020045

**Published:** 2023-06-02

**Authors:** Jane Tiller, Kristen Nowak, Tiffany Boughtwood, Margaret Otlowski

**Affiliations:** 1 Australian Genomics, Parkville, VIC 3052, Australia; 2 Murdoch Children’s Research Institute, Parkville, VIC 3052, Australia; 3 School of Public Health and Preventive Medicine, Monash University, Melbourne, VIC 3004, Australia; 4 Office of Population Health Genomics, Department of Health, Perth, WA 6004, Australia; 5 Centre for Law and Genetics, University of Tasmania, Hobart, TAS 7000, Australia

**Keywords:** privacy, genetics, ethics, genetic testing, cascade testing, medically actionable, risk notification, prevention

## Abstract

Genetic risk information has relevance for patients’ blood relatives. However, cascade testing uptake in at-risk families is <50%. International research supports direct notification of at-risk relatives by health professionals (HPs), with patient consent. However, HPs express concerns about the privacy implications of this practice. Our privacy analysis, grounded in a clinically relevant hypothetical scenario, considers the types of personal information involved in direct notification of at-risk relatives and the application of Australian privacy regulations. It finds that collecting relatives’ contact details, and using those details (with patient consent) to notify relatives of possible genetic risk, does not breach Australian privacy law, providing that HPs adhere to regulatory requirements. It finds the purported “right to know” does not prevent disclosure of genetic information to at-risk relatives. Finally, the analysis confirms that the discretion available to HPs does not equate to a positive duty to warn at-risk relatives. Thus, direct notification of a patient’s at-risk relatives regarding medically actionable genetic information, with patient consent, is not a breach of Australian privacy regulations, providing it is conducted in accordance with the applicable principles set out. Clinical services should consider offering this service to patients where appropriate. National guidelines would assist with the clarification of the discretion for HPs.

## 1. Introduction

Genetic risk information has relevance for patients’ blood relatives, especially for medically actionable conditions. Health professionals (HPs) discuss the importance of risk notification with patients and commonly provide “family letters” for distribution to at-risk relatives. However, the uptake of cascade testing in at-risk families is <50% [1]. A recent Australian study [2] found relatives had not been notified of genetic risk in >50% of families. The burden of contacting relatives was identified as a significant barrier to notification, especially for affected patients, indicating a need for supported communication. One mechanism to assist with increased cascade testing uptake is direct notification of at-risk relatives by HPs, ***with patients’ consent***. We note that disclosure of genetic results without patient consent is an important but separate topic, about which we have separately published [3].

The international literature supports the effectiveness of this practice, including strong public and patient support in multiple countries [1,4,5,6,7,8,9,10,11], with many studies recommending the consideration of direct contact of at-risk relatives by HPs. A 2022 systematic review and meta-analysis of 87 international studies found that direct contact increased the uptake of cascade genetic testing from 40% to 62% [12]. A 2016 Belgian study of *BRCA1/2* families found that direct notification almost doubled the cascade testing rate [4]. Australian studies about familial hypercholesterolaemia (FH) (genetic high cholesterol), show strong support from the public [13] and patients [14] for direct notification. A 2006 South Australian study also demonstrated a significant increase in cascade testing uptake for cancer variants after direct notification by HPs, and received no complaints about breach of privacy from individuals who were contacted directly [15]. A recently published study by authors of this manuscript also demonstrated strong support for direct notification amongst >1000 members of the Australian public, including very few privacy concerns [16].

Despite strong international evidence for the effectiveness and acceptability of this practice, Australian HPs anecdotally express concerns about its privacy implications. There are no published legal analyses of this practice from an Australian privacy perspective, or published national guidelines, to guide and inform HPs regarding their discretion and obligations in this area.

## 2. Materials and Methods

**Hypothetical case study**

**Figure 1:**
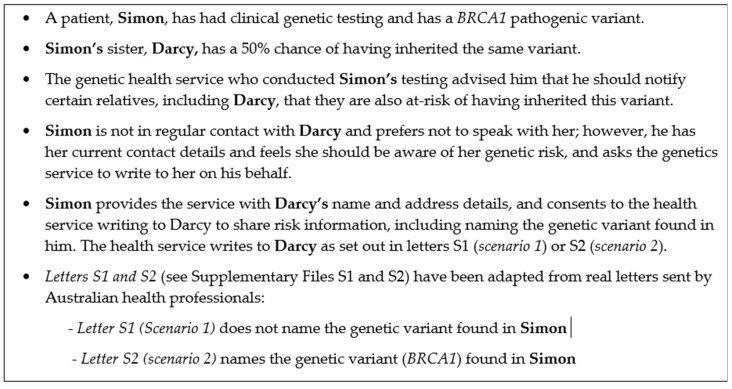
Case study (hypothetical).

The following hypothetical case study (Figure 1) is used as the basis for this privacy analysis.

**Legal analysis**

This analysis will answer the following questions:

1. What are the relevant Australian Commonwealth and state/territory privacy regulations?
2. Are the types of information collected and used in ***Letters S1/S2*** protected under privacy regulations?
3. Has the genetics service breached its privacy obligations by notifying **Darcy** directly in the hypothetical case study provided (Figure 1)?

This analysis is restricted to considering privacy implications of the collection, use and disclosure of personal information by HPs. It does not consider the impact of other regulations, such as restrictions on advertising or solicitation of business, that may apply to private HPs operating in a commercial setting.

## 3. Results

### 3.1. What Are the Relevant Australian Commonwealth and State/Territory Privacy Regulations?

The *Privacy Act 1988* (Cth) (PA) is the key privacy legislation applicable to HPs working in the private sector in Australia, and includes 13 Australian Privacy Principles (APPs). Relevant regulations also exist in all Australian states and territories (some of which have specific privacy regimes) and apply to HPs working in the public (and, sometimes, private) sector.

Table 1 sets out the various pieces of legislation and regulations that apply across various states and territories in Australia.

It is clear that where patients freely consent to use or disclosure of their own personal information, there is no breach of their privacy. This assumes that consent to the disclosure has been properly obtained. In Supplementary Files S1 and S2, **Simon** has consented to the disclosure of his information and it is reasonable to assume that consent was properly obtained. Accordingly, this analysis will focus on **Darcy’s** privacy.

### 3.2. Are the Types of Information Collected and Used in Letters S1/S2 Protected under Privacy Regulations?

**Figure 2:**
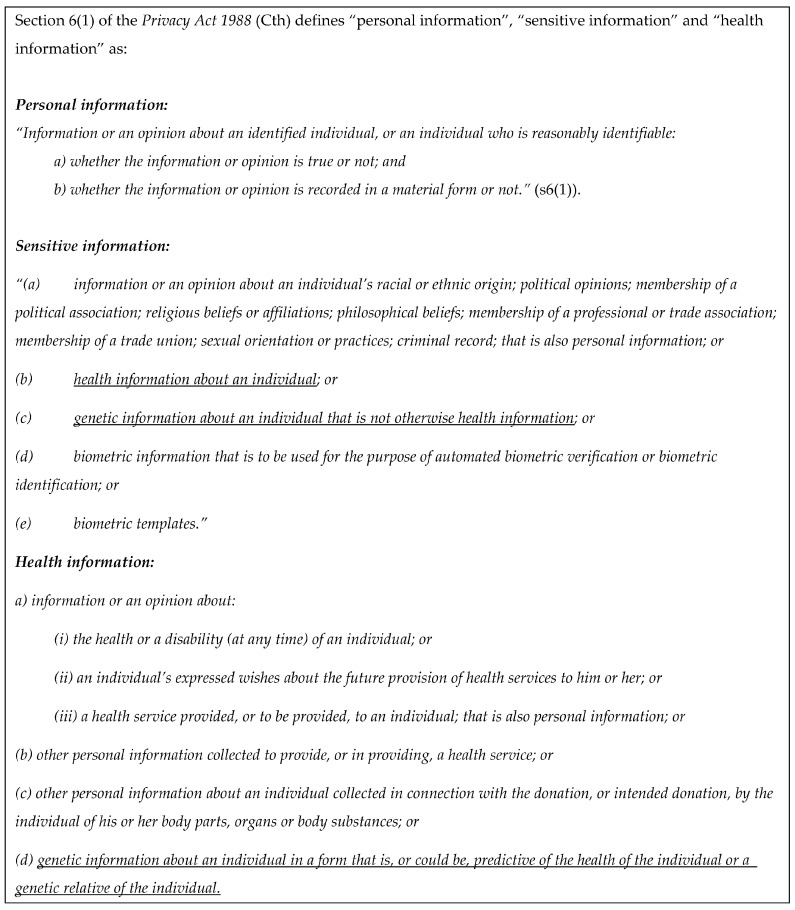
Definitions of personal information, sensitive information, and health information.

All “personal information” is protected under the PA. “Sensitive information” is a subset of personal information, and greater protection exists for information that is considered to be sensitive information (Figure 2). The State/Territory definitions are very similar.

There are two types of information that are being collected, used, and/or disclosed in this case study. The first type of information is **Darcy’s** contact details, and the second is the genetic information being included in the letter. This genetic information is both about **Simon’s** genetic status, and about **Darcy’s** possible genetic risk. In the discussion section we consider how the privacy regulations protect and regulate the use of this information.

The relevant Commonwealth APPs which apply to the collection, use, and/or disclosure of personal information in this context are **APP 3** (Collection of solicited personal information), **APP 5** (Notification of the collection of personal information) and **APP 6** (Use or disclosure of personal information). Table 2 summarizes these APPs and their application to this question, as well as listing the applicable state/territory principles. Although the language in the state/territory regulations is not identical, their effect is the same with a few notable exceptions. Those exceptions are noted in Table 2 and described, where applicable, below.

## 4. Discussion

***Has the genetics service breached its statutory privacy obligations by notifying Darcy directly in the hypothetical case study provided* (Figure 1)*?***

### 4.1. How Is the Use of Each Type of Information Identified in the Case Study Regulated by the Relevant Regulations?

#### 4.1.1. **Darcy’s** Contact Details

Individuals’ contact details, such as addresses and telephone numbers, are generally accepted to be personal information [17] (but not sensitive information), so their collection and/or use must comply with the requirements applicable to personal information.

#### 4.1.2. The Genetic Information

**Simon** has consented to the disclosure of his genetic information in both scenarios, so the question to be addressed is whether the genetic information in the letters (that **Darcy** is *at risk* of inheriting a familial variant) is (a) sensitive information and (b) genetic information *belonging to* **Darcy**. Given the definition of personal information (see Figure 2) includes information or an opinion about an identified individual, whether it is true or not, information that identifies an individual’s risk of developing disease appears to be personal information. The definition of “sensitive information” clearly includes genetic information (whether it is health information or not), so any genetic information about **Darcy** will also be sensitive information. 

Health information is defined to include, “*genetic information about an individual in a form that is, or could be, predictive of the health of the individual or a genetic relative of the individual*”. ***Letter S2*** includes specific information about the familial genetic condition, that seems to fall within this definition as it is in a form that could be predictive of the health of **Darcy** or her genetic relative (**Simon**). However, ***Letter S1***, which only refers generally to a relative having “a DNA change that increases the risk of developing an inherited medical condition”, is less obvious. Arguably, the fact that **Darcy** is at risk of inheriting an unnamed DNA variant is not specific enough to be health information about **Darcy** as it is not in a form that could be predictive of her health or her genetic relative’s health. 

The question, then, is whether **Darcy’s** risk of inheriting an unnamed DNA variant from an unnamed relative is genetic information (that is not health information) about her. The PA and explanatory material do not consider what constitutes genetic information that is not health information [18], and there is no judicial interpretation to assist. However, the 2006 amendment of the PA to insert genetic information that is not health information into the definition of sensitive information arose from the recommendations of the Australian Law Reform Commission and Australian Health Ethics report *Essentially Yours* (ALRC 96) [19]. These recommendations were intended to cover, for example, “genetic information derived from parentage or other identification testing that is not predictive of health”. 

The recent recommendations arising from the Australian Attorney General’s review of the PA, which recommend adding “genomic information” to the definition of sensitive information, do not further clarify this question [20]. This means that the information in ***Letter S2*** is likely to be personal and sensitive information about **Darcy**, whereas the information in ***Letter S1*** is likely to be personal information (as it is information about her), but it is unclear whether it is sensitive information. For this reason, it should be treated as sensitive information in this context to be prudent. Next, we will consider how the requirement that information must be about an “identified or reasonably identifiable” individual affects this categorization.

The definition of personal information (see Figure 2) applies to “Information or an opinion about an identified individual, or an individual who is reasonably identifiable”. Since (i) **Darcy** is identified and named and (ii) the familial genetic information is linked to her and used to inform the assessment of **Darcy’s** risk, the argument that the information contained in ***Letter S2*** is **Darcy’s** personal (and sensitive) information is further supported. However, the information about **Simon** would not be **Darcy’s** personal information without this additional link to her risk, as a reasonably identifiable individual she needs to be “a subject matter of the information or opinion” [21]. If information about **Simon’s** genetic variant on its own became **Darcy’s** personal information through the sharing of the information in either letter, this would raise the question of whether **Darcy** could control **Simon’s** sharing of his own genetic information without her consent.

It is clear that the parliamentary intention does not support an interpretation that **Darcy** could interfere with **Simon’s** sharing of his *own* genetic information. Although statutory interpretation must prioritize the word of the text, parliamentary materials may be used to provide context [22]. The Explanatory Memorandum for the *Privacy Legislation Amendment Bill 2006* (Cth) indicates that expressly including genetic information in the definitions (Figure 2) was intended to allow HPs’ discretion to advise relatives of genetic risk, even without patient consent [23]. However, this does not support an interpretation that Parliament intended to restrict individuals’ own autonomy with respect to their individual information. **Darcy** has no right to control the sharing of **Simon’s** personal information with others—that is **Simon’s** decision.

**In summary:**

- **Darcy’s** contact details are *personal information* and must be collected and used in accordance with the regulations applicable to personal information.
- The information contained in ***Letters S1 and S2*** is **Darcy’s** *personal information*.
- The genetic information contained in ***Letter S2*** (which names the specific gene) is likely to also be **Darcy’s** *sensitive information*, and must be used and/or disclosed in accordance with the regulations applicable to sensitive information.
- It is unclear whether the information contained in ***Letter S1*** (which does not name the specific gene and provides general information only) is *sensitive information*, but to be prudent it should be used and/or disclosed in accordance with the regulations applicable to sensitive information.
- **Simon’s** genetic information alone is not **Darcy’s** *personal information*.

Next, we consider whether the collection, use and/or disclosure of the personal information was a breach of privacy, or conducted in accordance with the relevant regulations (Table 2).

### 4.2. Are the Proposed Uses a Breach of Privacy?

We have concluded that **Darcy’s** contact details and the information in ***Letters S1*** and ***S2*** are *personal information*, and the genetic information in ***Letter S2*** (and potentially the information in ***Letter S1***) is *sensitive information* belonging to **Darcy**. The purpose of the collection and use of the contact details, and the use and disclosure of the genetic information in the letter, was to notify **Darcy** about her potential genetic risk. Facilitating the use of personal information to advise genetic relatives of their potential genetic risk was the primary reason behind the amendments which were made to the PA in 2006 to include genetic information in the PA framework [23].

#### 4.2.1. APP 3: Collection of Solicited Personal Information

APP 3 prohibits the collection of personal information unless reasonably necessary for the entity’s functions. Facilitation of cascade testing of at-risk relatives is a core function of genetics services [12,24,25,26,27,28,29,30,31,32], and communication of risk information to relatives by patients directly is frequently inadequate [2,29]. Accordingly, collecting **Darcy’s** personal information for this purpose sits squarely within its core functions. For sensitive information, APP 3 also requires the individual’s consent to collection. As contact details are not sensitive information, this aspect of APP 3 does not require **Darcy’s** consent for the collection of her contact information (although APP 5 requires her to be notified of certain things, as discussed below). This is consistent across all state/territory regulations other than in Western Australia (considered further below).

APP 3 also requires personal information be collected from individuals directly, unless unreasonable or impracticable to do so. The genetics service has no pre-existing relationship with **Darcy**, so collecting her contact details directly is clearly impracticable. Most states/territory regimes have similar effect, although Table 2 notes some differences in New South Wales and Western Australia. In New South Wales, s9 of the *Privacy and Personal Information Protection Act 1998* (NSW) (**PRIPA**) does not include the “unless unreasonable or impracticable’ exemption. However, s26(1) allows for an exemption where compliance would prejudice the interests of the individual to whom the information relates. Clearly, at-risk relatives’ interests will be prejudiced if they cannot be notified of their medically actionable genomic risk [12,24,25,32,33].

In Western Australia, which does not have a privacy regime, the collection, use, and disclosure of personal information are regulated under the *Health Services Act 2016* (WA), and is authorized if done with the consent of the person to whom it relates (s220(1)(a)). However, they can also be authorized under s220(1)(i) if any circumstances prescribed in the *Health Services (Information) Regulations 2017* (WA) apply. Under s5(1)(a) of those regulations, collection, use, or disclosure is authorized if reasonably necessary to lessen or prevent a serious risk to the life, health, or safety of an individual. Although genetic information is not explicitly mentioned in the WA Regulations, these are almost the exact words that were inserted into the PA to allow the disclosure of information to a genetic relative regarding their genetic risk [23]. This supports the conclusion that the WA regulations allow the collection of **Darcy’s** contact information from **Simon** without her consent, for the purpose of lessening or preventing a serious risk to her health, due to genetic risk.

Accordingly, in all jurisdictions, there is support for the argument that the collection of contact details without **Darcy’s** consent for the purposes of notification to her about her potential genetic risk is allowed.

#### 4.2.2. APP 5: Notification of Individuals

APP 5 requires entities who have collected personal information to take reasonable steps to notify individuals of matters including the entity’s contact details, the purpose of the collection (and any consequences flowing from not collecting the information), and mechanisms to complain about breach of privacy. These matters do not prevent the collection/use of contact details for risk notification, but must inform the content of any communication by HPs. ***Letters S1*** and ***S2*** have incorporated these requirements.

In addition to these Commonwealth PA obligations, which are largely mirrored by the various state/territory regulations, the Victorian Health Privacy Principles (HPP 1.7), require that reasonable steps are taken to ensure health information remains confidential when received from a recipient who is not the individual the health information is about. Some further general obligations to take reasonable steps to protect personal information are also found in the Commonwealth Australian Privacy Principles (APP 11.1); as well as those in Victoria (IPP 4.1/HPP 4.1); Australian Capital Territory (TPP 11/PP 4.1); New South Wales (HPP 5/PRIPA s12); Northern Territory (IPP 4.1); Queensland (IPP 4/NPP 4); South Australia (IPP 4(4)); and Tasmania (PIPP 4).

#### 4.2.3. APP 6: Use or Disclosure of Personal Information

APP 6 (and similar state/territory principles) limits use of **Darcy’s** personal information once collected. Personal information collected for one purpose (the primary purpose) cannot be used for another purpose (a *secondary purpose*) without consent, unless an exception applies. Adding **Darcy’s** contact details (without consent) to a mailing list, for example, would not be related to the primary purpose and would be a privacy breach. Contacting her as a follow-up to the letter that was sent would be related to the primary purpose, and would not be a breach of privacy unless she had expressly requested not to be contacted further.

The use of the genetic information about **Darcy** (her genetic risk) is also governed by APP 6. However, there is no breach of privacy in disclosing **Darcy’s** *own* personal information (her potential genetic risk) to her. The “right not to know” might be raised here to argue that it is a breach of **Darcy’s** rights to directly contact her with this information, without her consent. However, this purported “right” is not an element of statutory privacy obligations, or a right recognized under Australian privacy regimes. Rather, it is an ethical element (linked to autonomy) to be balanced against other elements (including the ethical imperative to provide access to medically actionable risk information) [34,35]. Because of the significant preventive potential of medically actionable risk information, the “right not to know” is significantly outweighed by the ethical imperative to offer this information to at-risk individuals [10].

Further, s16B(4) of the PA further supports disclosing to **Darcy** her potential genetic risk. Even *without* **Simon’s** consent, disclosure to **Darcy** is permitted if “necessary to lessen or prevent a serious threat to [her] life, health or safety”. A genetic predisposition to cancer has been specifically recognized as a serious threat to life, health, or safety, “*even where such a threat is not imminent*” [23]. Thus, the purported “right not to know” does not prevent the disclosure of this information directly to **Darcy**, especially with **Simon’s** consent. However, informing other entities or individuals of **Darcy’s** risk, without her consent, would be a privacy breach (unless another statutory exception applies). Furthermore, if **Darcy** asked not to be contacted further after the initial contact was made the HP should respect her wishes.

An important final point is that the discretion to contact at-risk relatives directly with patient consent, as discussed throughout this analysis, does not equate to a positive duty on HPs to contact relatives directly and notify them of their risk. No such obligation has been created in Australia, either through legislative instruments or Australian judicial findings. Rather, this analysis has confirmed that the discretion to do so (with the patient’s consent) exists, and is supported by the regulations governing HPs’ collection, use, and disclosure of personal information in all jurisdictions in Australia.

**In summary:**

- This analysis supports a conclusion that collection of **Darcy’s** contact details without her consent is allowed under all Australian privacy regulations, for the purpose of notifying her of her possible genetic risk.
- Reasonable steps should be taken to protect **Darcy’s** personal information once collected.
- **Darcy** should be notified as soon as possible after her contact details are collected, about the purpose of the collection and avenues to complain about breach of privacy.
- **Darcy’s** personal information (her contact details) can only be used for the primary purpose for which it was collected (to notify her about her possible genetic risk), not for any other purpose (without her consent).
- Disclosure of **Simon’s** genetic information to **Darcy** is permitted with his consent.
- Disclosure of **Darcy’s** *own* genetic information to her is permitted, and the purported “right not to know” does not prevent the disclosure of this information to **Darcy**, though her autonomy should be respected if she chooses not to pursue this further once notified.
- There is no positive duty on HPs to contact relatives directly to notify them of their risk—the discretion available to HPs to notify patients’ at-risk relatives directly is not an obligation.

## 5. Conclusions

Direct notification of patients’ at-risk relatives regarding medically actionable genetic information, with patient consent, is not a breach of Australian privacy law, providing it is conducted in accordance with the applicable regulatory principles as discussed throughout this analysis. Australian clinical services should consider offering direct notification of at-risk relatives to assist patients with family communication. This analysis provides an important resource for clinical services and HPs considering their obligations and discretion in this area; however, harmonized national guidelines would assist with the clarification of the discretion for HPs.

## Figures and Tables

**Table 1:** Commonwealth, State and Territory regulations relevant to collection, use and disclosure of personal information (applied in Table 2).

Jurisdiction	Act	Privacy Principles
**Commonwealth (CTH)**	*Privacy Act 1988* (Cth)	Schedule 1—Australian Privacy Principles (APP)
**Australian Capital Territory (ACT)**	*Information Privacy Act 2014* (ACT)	Schedule 1—Territory Privacy Principles (TPP)
	*Health Records (Privacy and Access) Act 1997* (ACT)	Schedule 1—Privacy Principles (PP)
**New South Wales (NSW)**	*Privacy and Personal Information Protection Act 1998* (NSW) (**PRIPA**)	N/A—applicable sections listed
	*Health Records and Information Privacy* Act 2002 (NSW)	Schedule 1—Health Privacy Principles (HPP)
**Northern Territory (NT)**	*Information Act 2002* (NT)	Schedule 2—Information Privacy Principles (IPP)
**Queensland (QLD)**	*Information Privacy Act* 2009 (QLD)	Schedule 3—Information Privacy Principles (IPP) and
		Schedule 4—National Privacy Principles (IPP)
**South Australia (SA)**	Premier and Cabinet Circular PC 012—Information Privacy Principles (IPPs) Instruction (2020)	Part II—Information Privacy Principles (IPP)
**Tasmania (TAS)**	*Personal Information Protection Act 2004* (TAS)	Schedule 1—Personal Information Protection Principles (PIPP)
**Victoria (VIC)**	*Health Records Act 2001* (VIC)	Schedule 1—Health Privacy Principles (HPP)
	*Privacy and Data Collection Act 2014* (VIC)	Schedule 1—Information Privacy Principles (IPP)
**Western Australia (WA)**	*Health Services Act 2016* (WA)	N/A—applicable sections listed
	*Health Services (Information) Regulations 2017* (WA)	N/A—applicable sections listed

**Table 2:** Application of privacy regulations to collection, use or disclosure of contact information and health information (see Table 1 for relevant regulations).

CTH Privacy Act	Clause	Application to Contact of At-Risk Relatives with Patient Consent	Principles in State/Territory Regulations Applicable to Collection, Use or Disclosure of Contact Information	Additional State/Territory Principles Applicable to Collection, Use or Disclosure of Health Information	Notes
**APP 3: Collection of solicited personal information**	3.2: Entity must not collect personal information (other than sensitive information) unless the information is reasonably necessary for one or more of the entity’s functions or activities.	Facilitating risk notification and cascade testing of relatives is one of the core functions of a clinical genetics service	ACT: TPP 3.1 NSW: PRIPA s8 NT: IPP 1.1 QLD: IPP1; NPP 1 VIC: IPP1.1 SA: IPP 4(1) TAS: PIPP 1		
	3.3: Entity must not collect sensitive information about an individual unless the individual consents to the collection of the information and the information is reasonably necessary for one or more of the entity’s functions or activities.	Personal contact details are not sensitive information, thus it is not necessary that relatives’ consent be obtained before the information is collected		VIC: IPP 10 ACT: TPP 3.3; PP 1 NSW: HPP 1 NT: IPP 10 TAS: PIPP 10	
	3.5: An APP entity must collect personal information only by lawful and fair means.	Collecting contact details of relatives directly from patients, with their consent, for the purpose of providing them with information about their genetic risk, is lawful and fair	VIC: IPP1.2 ACT: TPP 3.5 NSW: PRIPA s8 NT: IPP 1.2 QLD: IPP1 and NPP 1 SA: IPP 4(1) TAS: PIPP 1	VIC: HPP 1.2 NSW: HPP 1	
	3.6: An APP entity must collect personal information about an individual only from the individual unless it is unreasonable or impracticable to do so.	Given the purpose is to facilitate risk notification of relatives with whom the service has no contact, it is impracticable to collect contact details directly from those relatives	VIC: IPP 1.4 ACT: TPP 3.6 NSW: PRIPA s9 (and s26) NT: IPP 1.4 QLD: NPP 1 TAS: PIPP 1	VIC: HPP 1.2 NSW: HPP 1	NSW: PRIP s9 does not allow for exception to the requirement that personal information must be collected from the individual unless unreasonable or impracticable. However, s26(1) allows for an exemption where compliance would prejudice the interests of the individual to whom the information relates. Clearly, at-risk relatives’ interests will be prejudiced if they cannot be notified of their medically actionable genomic risk. WA: Collection, use or disclosure of personal information is authorised if done with the consent of the person to whom it relates (HSA s220(1)(a)). However, under HSIR s5(1)(a), collection, use or disclosure is authorised if reasonably necessary to lessen or prevent a serious risk to the life, health or safety of an individual.
**APP 5: Notification of the collection of personal information**	5.1 and 5.2: As soon as practicable after collecting personal information about an individual, the entity must take reasonable steps to notify the individual of the circumstances of the collection, the entity’s identity and contact details, the purpose of the collection and any consequences of not collecting the information, details of the entity’s privacy policy, mechanisms to correct information and avenues for complaints about breach of privacy, and any other bodies to which the information may be disclosed	These considerations should inform the content of the letter (or other form of communication) sent to relatives, but do not prevent the collection and use of the contact details for this purpose	VIC: IPP 1.3 and 1.5 ACT: TPP 5 NSW: PRIPA s10 NT: IPP 1.3 and 1.5 QLD: NPP 1 SA: IPP 4(2) TAS: PIPP 1	ACT: PP 2 VIC: HPP 1.4 and 1.5 NSW: HPP 4	VIC: HPP 1.7 requires that reasonable steps are taken to ensure that health information remains confidential where it is received from a recipient who is not the individual that the health information is about (for general obligations to take reasonable steps to protect personal information, see APP 11.1, VIC IPP 4.1/HPP 4.1; ACT TPP 11/PP 4.1; NSW HPP 5/PRIPA s12; NT IPP 4.1; QLD IPP 4/NPP 4; SA IPP 4(4); TAS PIPP 4).
**APP 6: Use or disclosure of personal information**	6.1: Personal information about an individual collected for a particular purpose (the *primary purpose*), must not be used or disclosed for another purpose (the *secondary purpose*) unless the individual consents or an exception applies	Contact details can only be used to contact relatives to notify them of their possible genetic risk and options for testing, not for any other purpose (without their subsequent consent)	VIC: IPP 2 ACT: TPP 6 NSW: PRIPA s17 NT: IPP 2.1 VIC: IPP10 QLD: NPP 2 SA: IPP 4(8) and IPP 4(10) TAS: PIPP 2	VIC: HPP 2 ACT: PP 9 and PP 10 NSW: HPP 1	VIC: IPP 1 applies to the use or disclosure of contact information (personal information that is not health information) VIC: IPP2 and HPP 2 applies to the use or disclosure of the health information. TAS: PIPP 9 has special provisions regarding the disclosure of personal information about an individual to an entity outside of Tasmania.

## Data Availability

There is no associated data.

## Supplementary Materials

The following supporting information can be downloaded at: https://www.mdpi.com/article/10.3390/biotech12020045/s1, Supplementary file S1: Scenario 1 Letter; Supplementary file S2: Scenario 2 Letter.
