# Supplementary material for: Privacy Implications of Contacting the At-Risk Relatives of Patients with Medically Actionable Genetic Predisposition, with Patient Consent: A Hypothetical Australian Case Study
Source: BioTech (Basel). 2023 Jun 2;12(2):45. doi: 10.3390/biotech12020045 (PMC10296322; doi:10.3390/biotech12020045)
Supplement: Supplementary file 1 [file biotech-12-00045-s001.zip › Supplementary file S1.pdf]

**Australian Clinical Genetics Service**

Dear [name]

I am a medical specialist with the Australian clinical genetics service. I am writing to tell you about important health information that is significant for you and your family members.

A biological relative of yours has been tested by our service and found to have a DNA change that increases the risk of developing an inherited medical condition. Your relative has asked that we share this information with you.

You may have inherited the DNA change found in your relative. If so, you will be able to access preventative options to avoid developing the condition, or to detect and treat it early. We have not included the name of the condition because you have not yet asked for this information for yourself.

If you have inherited this DNA change, you may also pass it on to any biological children you have/may have in the future.

You can have a genetic test (at no cost to you) to find out whether:

1) you have inherited the DNA change and need extra health care, or
2) you have not inherited the DNA change and do not need extra health care.

**We urge you to take this matter seriously.** This information could be very important for the health of you and your close relatives. We recommend that you contact us, or discuss this matter with your doctor within the next few months.

**What can you do if you would like a genetic test or have any questions?**

You can contact us by phone or email on (03) 99999999 or info@geneticsinfo.com with any questions or to discuss the next steps. Please quote the reference number at the top of this letter.

***If you do not want further information or an appointment at this stage, you are free to change your mind in the future. Please feel free to contact us at the above contact details at any time.***

Yours sincerely

Dr G Netix
Clinical Geneticist
Australian Clinical Genetics Service

***A note about privacy***

*Your relative provided us with your contact details so that we could give you this important information. We will only use your personal details for that purpose, and will not use them to contact you for other purposes. We will not provide your contact details to anyone else without your consent (unless required by law). We will delete or correct these details at your request, and you can contact us to request a copy of our privacy policy or to make a complaint about use of your information. Any information you provide to us will be treated confidentially. We will not tell anyone else, including your relatives, about any contact we may have with you without your permission. For the same reason, we cannot give you any information about other family members, including the relative who has provided us with your contact details.*
